# Behind the curtain: Workplace violence against nurses in Pakistan

**DOI:** 10.1016/j.amsu.2022.104378

**Published:** 2022-08-17

**Authors:** Imaan Ghalib Khan, Soha Abbas, Summaiyya Waseem

**Affiliations:** Dow University of Health Sciences, Pakistan

**Keywords:** Nurses, Violence, Healthcare, Ethics, WPV, Workplace violence, HCW, Healthcare workers

With severe consequences and grave detriments to workplace efficiency, workplace violence (WPV) is defined as incidents including assault, physical or mental abuse, and/or threats affecting the well-being and safety of an occupation [1]. Though regrettably, workplace violence(WPV) is familiar and known to all occupations and fields, the numbers in healthcare workers(HCWs) exceed by 16 times, ranking the healthcare sector as the leading and most exposed group affected by it [2,3].

Within the healthcare sector, nurses are estimated to be three times more affected by WPV than any other group [4], with 95% of them experiencing it at least once in their occupational setting [5]. Several studies have quoted regarding violence received by nurses in their profession. In recent literature, it was estimated that at least 16.5% of HCWs faced physical while 72.5% faced verbal assault, of which nurses made a total of a whopping 59% and 53% respectively [6]. Another study quoted that 73.1% of nurses encountered at least some type of WPV, with 53.4% suffering from physical, while 57.3% and 26.9% from verbal and sexual violence respectively [7].

Though numerous plausible reasons can be cited as causative factors; in almost two-thirds of the violent events, the perpetrators were either patients or their attendants [2]. Other factors leading to massive events against nurses include low professional status, poor perception of healthcare by the patient, unanticipated outcomes/death of a patient, resource constraints, delay in treatment, and a general lack of awareness in society. Another overlooked yet extremely important reason, lies in the fact that in Pakistan, nurses are predominantly female, making up to 95% of the task force. Hence, in male-dominant societies like Pakistan, where men tenant the leading job roles in the hospital hierarchy, WPV against female nurses is frequent [7]. Another key factor is the fact that most nurses perceive WPV to be a part of their job, hence the violence often goes unreported [7]. The incidents of WPV also vary depending on the location, with the emergency unit and wards within the facility being the center points for most of the incidents; each paying constituent to approximately one-third of the total events [2].

The abuse negatively impacts on different levels. On a personal level, i.e., nurses themselves suffer irritation, burnout, depression, nervousness, work-related stress, sleep disruption, panic attacks, a significant work-interest decline, and hence the fear of returning to work. A study in Jordan demonstrated that over half of the nurses who experienced WPV considered leaving their job [8]. In Pakistan, no proper study exists to calculate these numbers, however, owing to the massive percentage of WPV faced by this occupation, it can be estimated that many in this state would be considering the same. On an organizational level, the abuse manifest and reflects as increased absenteeism, decreased job performance and productivity, additional security costs, and workers’ compensation, thereby crashing the ultimate quality care provided [9]. Eventually, the patients are deprived of their deserved healthcare, in their time of dire need.

Addressing, tackling, and eventually eradicating the WPV prevalence against nursing staff is crucial yet challenging work for hospital management. On a personal level, before anything, it is imperative to emphasize on the fact that enduring WPV is not a part of a nurse's job, and any incident should be registered by them as well as brought up to the hospital management. Nurses should also receive adequate training to counter any assault incident or tricky situation. On an organizational level, a telecommunication project should be established, specifically working for nurses to call and lodge their complaints. Additionally, hospital security by installing cameras around the hospital and assigning chaperones to the nurses during their patient procedures should be done on a priority. On the governmental level, support groups should be launched to create safe spaces for nurses to talk about the problems they face during their work. Awareness-focused campaigns which can help educate nurses about laws protecting HCWs against WPV should be mass-delivered. Furthermore, the government should draft and introduce ethical guidelines to integrate a check and balance system with respect to each patient's case. Alongside, the government should introduce self-defense training programs to facilitate nurses to protect themselves from any further attacks. Reforms to protect HCWs should be renewed, considering new cases and strict punishment should be announced for anyone found guilty of committing a violent act. Moreover, allocating an appropriate healthcare budget to reduce the crowd, and dissatisfaction of patients should be taken under consideration. It's high time diligent measures are taken, or else if this trend persists, outmigration of nurses from the country will rise and the subsequent performance of the health sector in Pakistan will deteriorate [10].

## Please state any conflicts of interest

The authors declare that there is no conflict of interest.

## Please state any sources of funding for your research

No funding was acquired for this paper.

## Ethical approval

The paper did not involve patients; therefore no ethical approval was required.

## Author contribution

Imaan Ghalib Khan: conception of the study, drafting of the work, final approval and agreeing to the accuracy of the work. Soha Abbas: conception of the study, drafting of the work, final approval of the work and agreeing to the accuracy of the work. Summaiyya Waseem: drafting of the work, final approval and agreeing to the accuracy of the work.

## Consent

The paper did not involve patients, therefore no consent was required.

## Registration of research studies

• Name of the registry: N/A
• Unique Identifying number or registration ID: N/A
• Hyperlink to your specific registration (must be publicly accessible and will be checked): N/A

## Guarantor

Imaan Ghalib Khan.
